# Handgrip exercise does not alter CO_2_‐mediated cerebrovascular flow‐mediated dilation

**DOI:** 10.1113/EP091125

**Published:** 2023-08-25

**Authors:** Shotaro Saito, Hironori Watanabe, Erika Iwamoto, Shigehiko Ogoh

**Affiliations:** ^1^ Department of Biomedical Engineering Toyo University Kawagoe Japan; ^2^ School of Health Science Sapporo Medical University Sapporo Japan; ^3^ Neurovascular Research Laboratory University of South Wales Pontypridd UK

**Keywords:** cerebral endothelial function, cerebrovascular shear rate, executive function, internal carotid artery, isometric exercise

## Abstract

Handgrip exercise (HG), a small muscle exercise, improves cognitive function and is expected to provide a useful exercise mode to maintain cerebral health. However, the effect of HG on cerebral blood flow regulation is not fully understood. The present study aimed to examine the effect of acute HG on cerebral endothelial function as one of the essential cerebral blood flow regulatory functions. Thirteen healthy young participants performed interval HG, consisting of 4 sets of 2 min HG at 25% of maximum voluntary contraction with 3 min recovery between each set. Cognitive performance was evaluated before and at 5 and 60 min after interval HG using the Go/No‐Go task (reaction time and accuracy). The diameter and blood velocity of the internal carotid artery (ICA) were measured using a duplex Doppler ultrasound system. To assess cerebral endothelial function, hypercapnia (30 s of hypercapnia stimulation, end‐tidal partial pressure of CO_2_: +9 mmHg)‐induced cerebrovascular flow‐mediated dilatation (cFMD) was induced, calculated as relative peak dilatation from baseline diameter. The shear rate (SR) was calculated using the diameter and blood velocity of the ICA. As a result, cognitive performance improved only at 5 min after interval HG (reaction time, *P* = 0.008; accuracy, *P* = 0.186), whereas ICA SR during interval HG and cFMD after interval HG were unchanged (*P* = 0.313 and *P* = 0.440, respectively). These results suggest that enhancement in cerebral endothelial function is not an essential mechanism responsible for acute HG‐induced cognitive improvement.

## Abstract (highlights)

- **What is the central question of this study?**
  Does handgrip exercise, a small muscle exercise, improve cerebral endothelial function?
- **What is the main finding and its importance?**
  Acute interval isometric handgrip exercise (2 min of exercise at 25% maximum voluntary contraction, followed by 3 min of recovery, repeated for a total of 4 sets) did not improve cerebral endothelial function. Since the cerebrovascular shear rate did not change during exercise, it is possible that acute handgrip exercise is not sufficient stimulation to improve cerebral endothelial function.

## 1 INTRODUCTION

The epidemiological studies have demonstrated that habitual exercise reduces the risk of dementia and stroke (Bliss et al., 2021; Chang et al., 2012; Cui et al., 2018; Gallanagh et al., 2011), therefore, making exercise therapy a highly recommended initiative to maintain brain health. Although exercise therapy using large muscles is effective for improving cognitive function in elderly and patient populations, these individuals often have difficulty performing this mode of exercise. In this regard, small muscle exercises such as handgrip exercises (HG) are a convenient mode of exercise therapy for the elderly and patient populations (e.g., bedridden patients), because the exercises can be performed in any position or location (e.g., a hospital bed). Indeed, our recent study (Washio et al., 2021) demonstrated that interval HG improves cognitive performance, indicating that HG is also a useful mode for enhancing cognitive function. However, its physiological mechanism remains unknown.

One possible mechanism by which HG improved cognitive function is cerebral endothelial function. In cerebral circulation, endothelial function is involved in regulating cerebral blood flow (CBF) to maintain cerebral homeostasis (Toda et al., 2009; White et al., 2000). Indeed, it is suggested that impairment of CBF regulation with endothelial dysfunction is a risk factor for cerebral disease (e.g., dementia) (de la Torre, 2012; de la Torre & Aliev, 2005; Toda et al., 2009; Wolters et al., 2016, 2017). Therefore, cerebral endothelial function may be the key factor in determining cognitive function. It is well established that the endothelial function in the peripheral circulation is improved by increasing blood flow, more specifically, by increasing vascular shear rate (SR) (Tinken et al., 2009). Similarly, cerebral endothelial function is possibly improved by an exercise‐induced increase in CBF or cerebrovascular SR (Ogoh & Ainslie, 2009; ogoh et al., 2021; Smith et al., 2019). Under these background, in the present study, we hypothesized that HG enhances cerebral endothelial function and consequently improves cognitive performance. To test our hypothesis, we evaluated cognitive performance and 30 s of hypercapnia stimulation‐induced flow‐mediated dilatation (FMD) in the internal carotid artery (ICA) as an index of cerebral endothelial function (cerebrovascular FMD; cFMD) (Carr et al., 2020; Hoiland et al., 2017, 2022) before and after interval HG.

## 2 METHODS

### 2.1 Ethical approval

The protocol was approved by the Institutional Review Board of Toyo University (Approval Number: TU2021‐028) and conformed to the standards set by the *Declaration of Helsinki*, except for registration in a database. All participants provided written informed consent before participation. Participants were made aware of the intent to publish these data when providing informed consent, and participants cannot be individually identified from data published in this paper.

### 2.2 Participants

Thirteen healthy participants (10 males and 3 females; mean age, 21 ± 1 years; height, 167 ± 7 cm; weight, 62 ± 12 kg; and body mass index, 22 ± 3 kg/m^2^) were recruited in this study. None of the participants had any known cerebrovascular or cardiovascular disease, they were not taking medications and they were non‐smokers. Before the experiment, each participant was required to abstain from caffeine, alcohol and strenuous exercise for 24 h. Furthermore, the experiment was performed at least 6 h after a light meal. Experiments were conducted between 09.00 and 12.00 h (*n* = 7) or 13.00 and 16.00 h (*n* = 6).

### 2.3 Experimental procedure

Within a week prior to the experiment, maximum muscle strength was measured using maximal voluntary isometric handgrip contraction (MVC) of the left hand (non‐dominant arm) to determine the exercise intensity of the interval HG. The MVC value was adopted as the highest force produced during three maximal efforts, each separated by 1 min. All participants then completed at least one practice of the Go/No‐Go task (30 trials) to minimize learning effects.

On the day of the experiment, the Go/No‐Go task and cFMD were measured in this order before performing interval HG (Pre), and then the participants performed left‐hand interval HG using visual feedback. These measurements were repeated 5 and 60 min after the interval HG (Post5 and Post60, respectively). The interval HG consisted of 4 sets of 2 min HG at 25% MVC with 3 min recovery between each set, which has been reported to enhance cognitive performance (Washio et al., 2021). The room temperature was set at 24−25°C.

To confirm that the current experimental protocol would not have a learning effect on cognitive performance, a protocol without exercise (control condition) was performed using other participants (*n* = 13, 8 males and 5 females; mean age, 22 ± 1 years) and the Go/No‐Go was measured at Pre, Post5 and Post60.

### 2.4 Measurements

#### 2.4.1 Cardiorespiratory measurements

Heart rate (HR) was measured using a lead II electrocardiogram (Bedside monitor BMS‐3400; Nihon Kohden, Tokyo, Japan). Beat‐to‐beat arterial blood pressure (ABP) was monitored continuously using finger photoplethysmography (Finometer Pro; Finapres Medical Systems, Amsterdam, Netherlands) with a cuff placed on the middle finger of the right hand. To calibrate beat‐to‐beat ABP, the systolic and diastolic blood pressure values in the brachial artery were measured (Tango +; SunTech Medical, Eynsham (Witney), UK). Minute ventilation (${\dot{V}}_{E}$), tidal volume (*V*_t_), respiratory rate, and end‐tidal partial pressure of CO_2_ (*P*_ET_CO_2_) were measured breath‐by‐breath using an automated gas analyzer (AE‐310S; Minato Medical Science, Osaka, Japan). All data were sampled continuously at 1 kHz using an analog‐to‐digital converter (PowerLab 16S; ADInstruments, Sydney, Australia) and stored on a laboratory computer for offline analysis.

#### 2.4.2 Cerebrovascular measurements

The right ICA diameter and time‐averaged mean blood velocity were measured using duplex Doppler ultrasound system (Vivid i; GE Healthcare, Chicago, IL, USA) equipped with 13‐MHz linear array transducers. The right ICA longitudinal image (i.e., diameter) and time‐averaged mean blood velocity were obtained using B (brightness) and pulsed‐wave modes, respectively. The right ICA was imaged in 1.0−1.5 cm distal to the carotid bifurcation. The position of the linear array transducer was kept constant at an insonation angle < 60°. These ICA variables were recorded at 30 Hz as a video file using a video capture device (DVI2USB 3.0; Epiphan Systems, Ottawa, Canada) for offline analysis.

#### 2.4.3 Cognitive performance (Go/No‐Go task)

Cognitive performance was assessed using the Go/No‐Go task (Presentation ver. 21; Neurobehavioral Systems Inc., Albany, CA, USA) which requires executive function (Akagi et al., 2019; Saito et al., 2021; Washio et al., 2021). In this task, a preparatory stimulus (a green square) was displayed for 1 s on the computer screen in front of the participants. Then, the target stimuli (a red and a blue square) or non‐target stimuli (a yellow and a purple square) were displayed for 1 s. If the target stimuli were displayed, the participants were asked to press the left button of the computer mouse with their right index finger as quickly as possible. If non‐target stimuli were displayed, the participants were asked to refrain from responding. This task consisted of 60 trials with equal probability (30 target stimuli and 30 non‐target stimuli). To evaluate cognitive performance, the mean reaction time to target stimuli and the accuracy of 60 trials in the Go/No‐Go task were calculated.

#### 2.4.4 Cerebral endothelial function (cFMD test)

**FIGURE 1:**
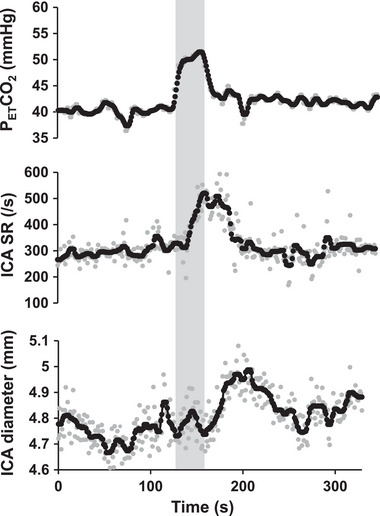
The average 1 s response for end‐tidal carbon dioxide (*P*_ET_CO_2_), shear rate (SR), and diameter in the internal carotid artery (ICA) during the cerebrovascular flow‐mediated dilatation (cFMD) test in a typical individual. Grey circles represent raw data, and black circles represent smoothed data. The grey areas indicate the period during which transient CO_2_ inhalation (+9 mmHg *P*_ET_CO_2_) was applied.

Cerebral endothelial function was characterized by transient hypercapnia intervention (for 30 s)‐induced shear‐mediated ICA vasodilatation response (i.e., cFMD, Figure 1) based on previous studies (Carr et al., 2020; Hoiland et al., 2017, 2022). The validity of this method for assessing cerebral endothelial function was confirmed by a previous study showing administration of nitric oxide synthase (NOS) inhibitor attenuates the ICA dilatation (Hoiland et al., 2022). In the cFMD test, we measured the right ICA diameter and blood velocity from 2 min resting baseline to 3 min after the onset of 30 s of transient hypercapnia stimulations. Since maximal vasodilatation has been observed to occur within 3 min after CO_2_ inhalation in young subjects (Hoiland et al., 2021), we employed a shorter Doppler measurement duration (3 min after the onset of CO_2_ inhalation) than in the original transit CO_2_ test studies (Carr et al., 2020; Hoiland et al., 2022, 2017). During hypercapnia, participants inhaled mixed gas (room air and 100% CO_2_) using a mixing chamber (250 ml gas blender; Arco System, Chiba, Japan) and *P*_ET_CO_2_ was quickly (∼5 s) elevated to +9 mmHg from resting baseline by manually adjusting CO_2_ concentration in mixing chamber. During the cFMD test, the participants were paced at 20 breaths/min using a metronome and *V*_t_ was simultaneously adjusted using visual feedback to keep ${\dot{V}}_{E}$ unchanged. This instruction was utilized to quickly and adequately manipulate *P*_ET_CO_2_ values during the cFMD test because a higher respiratory rate allows for quicker breath‐by‐breath gas exchange (Carr et al., 2020; Hoiland et al., 2017, 2022).

### 2.5 Data analysis

#### 2.5.1 Cardiorespiratory measurements

Mean arterial pressure (MAP) was obtained from the ABP waveform. HR, MAP, ${\dot{V}}_{E}$ and *P*_ET_CO_2_ were analysed offline using signal processing software (LabChart 8, ADInstruments). The data during the interval HG were averaged for the last minute of HG and the recovery of each set. In addition, these data during the cFMD test were averaged for 2 min of resting baseline and 30 s of transient hypercapnia stimulations.

#### 2.5.2 Cerebrovascular measurements during exercise

ICA diameter and time‐averaged mean blood velocity were analysed using custom‐designed edge‐detecting and wall‐tracking software (version 2.0.4, S‐13037; Takei Scientific Instruments, Niigata, Japan). ICA SR and blood flow were calculated from the measurement of ICA diameter and blood velocity (SR (/s) = 4 (blood viscosity constant) × blood velocity/diameter; and blood flow (ml/min) = π × (diameter/2)^2^ × blood velocity × 60). In addition, ICA conductance (ml/min/mmHg) was calculated as blood flow divided by MAP. These data were averaged for the last minute of HG and the recovery of each set in the interval HG.

#### 2.5.3 cFMD test

ICA diameter and time‐averaged mean blood velocity were analysed using custom‐designed edge‐detecting and wall‐tracking software (version 2.0.4, S‐13037). Missing ICA diameter data (6.4%) during the cFMD test due to technical issues or swallowing were interpolated by the least squares interpolation method, and then these ICA data were resampled to 1 Hz and filtered using the two‐stage filtering process (median filter and Savitzky–Golay finite impulse response smoothing filter) (Carter et al., 2016; Sakamoto et al., 2021). The median values of the diameter and SR during resting baseline were detected as the ICA baseline diameter (*D*_base_) and SR (SR_base_). The peak values of diameter and SR for 3 min after transient hypercapnia stimulations were detected as ICA peak diameter (*D*_peak_) and SR (SR_peak_). The cFMD was calculated using the following equation: cFMD (%) = (*D*_peak_ − *D*_base_)/*D*_base_ × 100. The SR area under the curve (SR_AUC_) was quantified as the area from the onset of hypercapnia to the time of *D*_peak_ and calculated using the trapezoidal rule as SR_AUC_ (a.u.) = Σ[1/2 (*x_i_*_+1_ − *x_i_*) (*y_i_*_+1_ − *y_i_*) + (*x_i_*_+1_ − *x_i_*) (*y_i_* − *z*)], where *x* is time, *y* is SR, and *z* is SR_base_. One participant was excluded from cFMD data analysis because ICA diameter could not be measured due to technical issues.

#### 2.5.4 Statistics analysis

All data were expressed as median (interquartile range; IQR) or mean (standard deviation; SD) and analysed using statistical software (SPSS Statistics Ver. 27, IBM, Tokyo, Japan). The Shapiro–Wilk test and Leven test were applied to verify the normal distribution and the equal variance, respectively, for each measurement. Cardiorespiratory responses during transient hypercapnia stimulation of the cFMD test were evaluated using a two‐way repeated‐measures analysis of variance (ANOVA, Time × CO_2_). For all other data, normally distributed data and equal variance data were analysed using a one‐way repeated‐measure ANOVA with Bonferroni correction for multiple comparisons, and the non‐normally distributed or unequal variance data were assessed using non‐parametric ANOVA (Friedman's test) with Wilcoxon's matched‐pairs test. To confirm that the current experimental protocol would not cause a learning effect, we performed two‐way mixed design ANOVA (Time × Condition) including data of protocol without exercise (control condition, *n* = 13) to evaluate the effect of interval HG on cognitive performance (reaction time and accuracy). To consider differences in SR_AUC_ before and after exercise, we performed an analysis of covariance (ANCOVA) with SR_AUC_ as a covariate to evaluate cFMD (corrected‐cFMD). Statistical significance was set at *P <* 0.05.

## 3 RESULTS

### 3.1 Cardiorespiratory and cerebrovascular responses during interval HG

HR and MAP increased during HG of the 2nd, 3rd and 4th sets and of all sets, respectively (*P* < 0.050 and *P* < 0.007, respectively, Table 1), and then decreased to comparable resting baseline values during recovery (*P* = 1.000 for all). ${\dot{V}}_{E}$ and *P*_ET_CO_2_ did not change compared with the resting baseline during all sets of HG (*P* = 1.000 and *P* > 0.459, respectively).

**TABLE 1:** Cardiorespiratory and cerebrovascular response during interval handgrip exercise.

		1st set		2nd set		3rd set		4th set		
	Baseline	HG	Recovery	HG	Recovery	HG	Recovery	HG	Recovery	*P* values (main effect of time)
HR (beats/min)	66 (58–71)	71 (63–74)	65 (60−75)	73 (63−77)*	68 (61−75)	74 (68−79)*	68 (63−73)	77 (68−81)*	67 (63−70)	<0.001
MAP (mmHg)	93 (91−100)	102 (97−105)*	94 (89−100)	102 (100−107)*	94 (88−99)	106 (99−110)*	95 (88−101)	109 (101−111)*	96 (88−101)	<0.001
${\dot{V}}_{E}$ (l/min)	8.5 (7.6−9.3)	8.8 (8.2−10.5)	8.8 (8.2−9.6)	9.0 (7.8−9.8)	8.5 (8.1−8.7)	9.1 (8.0−10.3)	8.5 (8.2−8.8)	9.4 (7.4−10.4)	8.2 (7.6−8.8)	0.010
*P*_ET_CO_2_ (mmHg)	36.3 (34.6−38.0)	37.0 (35.1−38.4)	36.1 (34.6−36.8)	36.4 (34.4−38.6)	35.9 (33.9−38.2)	36.6 (34.3−37.5)	35.4 (34.0−38.1)	35.9 (32.8−38.0)	36.4 (33.9−38.1)	0.001
ICA diameter (mm)	5.35 (4.91−5.40)	5.49 (5.10−5.71)*	5.31 (5.02−5.44)	5.52 (5.20−5.78)*	5.39 (5.08−5.54)	5.61 (5.05−5.67)*	5.37 (4.81−5.55)	5.46 (4.84−5.80)*	5.20 (4.80−5.54)	<0.001
ICA blood velocity (cm/s)	28.2 (27.6−29.7)	30.0 (27.9−31.5)	27.8 (25.5−29.0)	28.7 (26.2−29.7)	26.9 (25.4−28.7)	27.7 (26.8−29.0)	27.4 (27.0−28.2)	29.9 (25.6−30.6)	28.3 (25.1−30.1)	0.054

All values are median (IQR). HR, MAP and *P*_ET_CO_2_ were evaluated using a one‐way repeated‐measures ANOVA with Bonferroni correction for multiple comparisons. ${\dot{V}}_{E}$, ICA diameter and ICA blood velocity were evaluated using non‐parametric ANOVA (Friedman's test) with Wilcoxon's matched‐pairs test. *P < 0.05 vs. resting baseline. HG, handgrip exercise; HR, heart rate; ICA, internal carotid artery; MAP, mean arterial pressure; *P*_ET_CO_2_, end‐tidal partial pressure of CO_2_; ${\dot{V}}_{E}$, minute ventilation.

**FIGURE 2:**
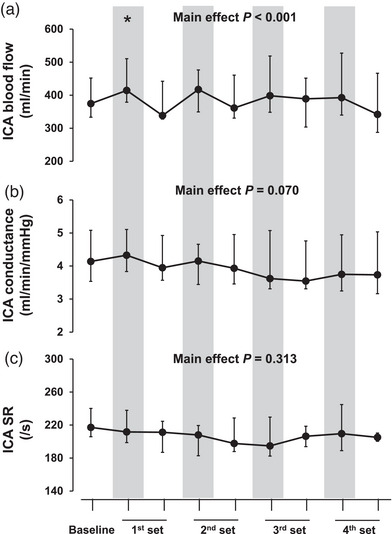
Blood flow (a), conductance (b), and shear rate (SR; c) in the internal carotid artery (ICA) during interval handgrip exercise (HG). The grey areas indicate duration of the HG of each set. All values are median (IQR). *n* = 13. ^*^ *P* < 0.05 vs. baseline.

During HG of all sets, the ICA diameter increased compared with the resting baseline (*P* < 0.037, Table 1), and then returned to baseline values during all recovery phases (*P* > 0.110). ICA blood flow increased only during HG of the 1st set (*P* = 0.015, Figure 2a). However, ICA blood velocity, conductance and SR did not change throughout the interval HG (*P* > 0.050 for all, Table 1, Figure 2b,c).

### 3.2 Cognitive performance

The reaction time in the Go/No‐Go task was shorter after the interval HG at Post5 compared with Pre (Pre vs. Post5, 398 (380−424) ms vs. 376 (367−398) ms, *P* = 0.008, Table 2), and then returned to Pre value at Post60 (Pre vs. Post60, 398 (380−424) ms vs. 398 (367−418) ms, *P* = 0.595), but did not change in the control condition (Pre vs. Post5 and Pre vs. Post60, *P* = 1.000 for all). The accuracy in the Go/No‐Go task was not changed by either the interval HG or control condition (*P* = 0.186, Table 2). These results indicate that the learning effect under the present protocol was minimal.

**TABLE 2:** Cognitive performance before and after interval handgrip exercise.

	Control			Interval handgrip exercise			*P* values (two‐way mixed design ANOVA)		
	Pre	Post5	Post60	Pre	Post5	Post60	Time	Condition	Interaction
Reaction time (ms)	386 (341−397)	374 (339−407)	387 (340−398)	398 (380−424)	376 (367−398)*	398 (367−418)	0.201	0.234	0.029
Accuracy (%)	100 (100−100)	100 (98−100)	100 (98−100)	100 (98−100)	100 (100−100)	100 (100−100)	0.529	0.352	0.186

All values are median (IQR). The reaction time and accuracy in the go/no‐go task were evaluated using a two‐way mixed design ANOVA (Time × Condition) with Bonferroni correction for multiple comparisons. *P < 0.05 vs. Pre. Pre, before interval handgrip exercise (HG); Post5, at 5 min after interval HG; Post60, at 60 min after interval HG.

#### 3.2.1 cFMD test

**FIGURE 3:**
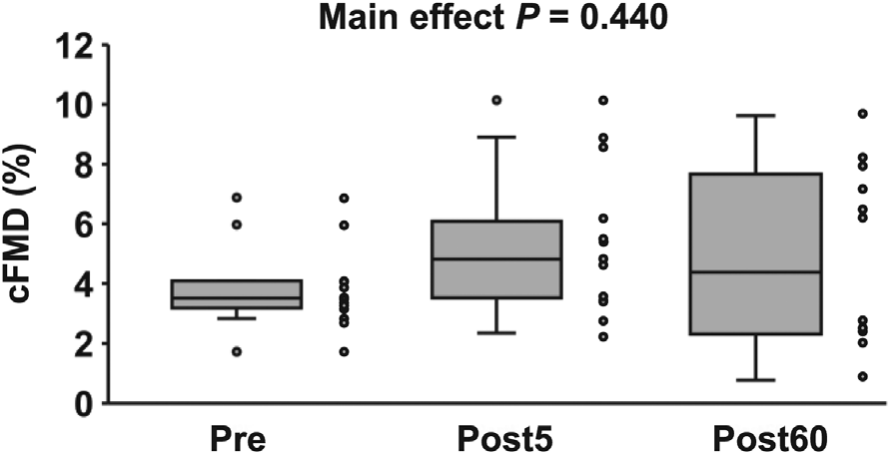
Cerebrovascular shear‐mediated dilatation (cFMD) before (Pre) and 5 and 60 min after interval handgrip exercise (HG) (Post5 and Post60, respectively). Black circles represent individual data. All values are median (IQR and max/min). *n* = 12.

The absolute changes in *P*_ET_CO_2_ from resting baseline to transient hypercapnia stimulations were not different in each cFMD test (Pre vs. Post5 vs. Post60, +9.5 (9.2−10.2) vs. +9.7 (9.1−9.9) vs. +9.6 (8.9−10.2) mmHg, *P* = 0.761). Therefore, transient hypercapnia stimulation was comparable in each cFMD test (*P* = 0.715, Table 3). *D*_peak_ was detected within 3 min after the onset of transient hypercapnia stimulation and the ICA diameter gradually decreased after this time point (Table 4). ICA *D*_base_, SR_base_, time to peak dilatation, *D*_peak_, SR_peak_ and SR_AUC_ did not change before and after the interval HG (*P* > 0.050 for all, Table 4). Interval HG did not change cFMD (*P* = 0.440, Figure 3). When adjusting cFMD using SR_AUC_, the corrected cFMD also did not change via interval HG (*P* = 0.208, Table 4).

**TABLE 3:** Cardiorespiratory response during cerebrovascular flow‐mediated dilatation test.

	Pre		Post5		Post60		*P* values (two‐way ANOVA)		
	Baseline	CO_2_	Baseline	CO_2_	Baseline	CO_2_	Time	CO_2_	Interaction
HR (beats/min)	59 (56−66)	61 (58−69)	59 (57−65)	62 (58−67)	59 (55−64)	63 (56−67)	0.146	0.417	0.067
MAP (mmHg)	89 (78−91)	90 (75−93)	88 (85−97)	88 (85−95)	87 (82−92)	85 (78−94)	0.781	0.291	0.098
*P*_ET_CO_2_ (mmHg)	39.4 (36.3−39.9)	49.1 (47.1−50.1)	39.5 (36.6−40.1)	48.3 (46.1−49.8)	39.3 (36.9−39.7)	48.3 (46.3−49.9)	0.403	< 0.001	0.715

All values are median (IQR). These data were evaluated using a two‐way repeated‐measures ANOVA (Time × CO_2_). Pre, before interval handgrip exercise (HG); Post5, at 5 min after interval HG; Post60, at 60 min after interval HG; CO_2_, during transient hypercapnia; HR, heart rate; MAP, mean arterial pressure; *P*_ET_CO_2_, end‐tidal partial pressure of CO_2_.

**TABLE 4:** Vascular response in the internal carotid artery during cerebrovascular flow‐mediated dilatation test.

	Pre	Post5	Post60	*P* values (main effect of time)
*D*_base_ (mm)	4.85 (4.68−5.36)	4.92 (4.71−5.32)	4.86 (4.64−5.31)	0.175
*D*_peak_ (mm)	4.98 (4.84−5.60)	5.15 (4.90−5.57)	5.25 (4.93−5.62)	0.706
Time to peak dilatation (s)	108 (87−108)	88 (64−125)	109 (79−141)	0.545
SR_base_ (/s)	296.5 (278.7−348.0)	281.0 (227.1−319.3)	267.0 (250.9−312.2)	0.205
SR_peak_ (/s)	384.2 (341.7−441.0)	326.5 (295.8−408.2)	345.2 (316.7−387.1)	0.324
SR_AUC_ (a.u.)	2144 (1696−3059)	2401 (1413−3645)	2730 (1771−3911)	0.758
Corrected‐FMD (%)	3.8 (2.4)	5.5 (2.4)	4.8 (2.5)	0.208

All values are median (IQR) or mean (SD). Time to peak dilatation, SR_base,_ and SR_peak_ were evaluated using a one‐way repeated‐measures ANOVA with Bonferroni correction for multiple comparisons. *D*_base_, *D*_peak,_ and SR_AUC_ were evaluated using non‐parametric ANOVA (Friedman's test) with Wilcoxon's matched‐pairs tests. Corrected‐FMD were evaluated using an ANCOVA with SR_AUC_ as a covariate. Pre, before interval handgrip exercise (HG); Post5, at 5 min after interval HG; Post60, at 60 min after interval HG; *D*_base_, baseline diameter in the internal carotid artery (ICA); *D*_peak_, peak diameter in ICA; SR_AUC_, shear rate area under the curve; SR_base_, baseline shear rate in the ICA; SR_peak_, peak shear rate in the ICA.

## 4 DISCUSSION

The present study for the first time examined whether small muscle exercise (HG) improves cerebral endothelial function, similar to cognitive function. As expected, the interval HG protocol improved cognitive performance, however, cerebral endothelial function was unchanged and there were no changes in cerebrovascular SR during exercise. These findings suggest that interval HG improved cognitive function but this exercise procedure is not sufficient stimulation for improving cerebral endothelial function. These findings suggest that enhancement in cerebral endothelial function is not an essential mechanism responsible for acute HG‐induced cognitive improvement.

Exercise therapy is recommended as one of the initiatives for maintaining cerebral health because it is well‐documented that habitual exercise reduces the risk of dementia and stroke (Bliss et al., 2021; Chang et al., 2012; Cui et al., 2018; Gallanagh et al., 2011). However, the direct effects of exercise on cerebrovascular function (e.g., endothelial function) are not well understood. For these reasons, some studies have investigated the acute effect of dynamic exercise on hypercapnia‐induced dilatation in the ICA (Sakamoto et al., 2021, 2022). Sakamoto et al. reported that high‐intensity aerobic exercise attenuated the hypercapnia‐induced dilatation in the ICA at 5 min post‐exercise and returned to baseline value at 60 min post‐exercise, but moderate‐intensity aerobic exercise did not affect it (Sakamoto et al., 2021). Accordingly, there is still no evidence that acute exercise can improve cerebrovascular function (Sakamoto et al., 2021, 2022). In contrast, Iwamoto et al. reported that intermittent hypoxia enhances hypercapnia‐induced dilatation in the ICA at 25 min post‐intervention (Iwamoto et al., 2020). Regarding the kinetics of the changes of cFMD, we expected that HG would cause acute (within the 60 min) changes in cerebral endothelial function in the present study from the findings of these previous studies (Iwamoto et al., 2020; Sakamoto et al., 2021) and thus we measured cFMD at 5 min and 60 min after HG. However, in the present study, cFMD did not improve following HG (Figure 3).

Elevation of vascular SR is a key factor in improving vascular endothelial function (Iwamoto et al., 2020; Tinken et al., 2010). Indeed, it has been reported that aerobic exercise, rhythmic HG and heating increased peripheral vascular SR and enhanced peripheral FMD (Tinken et al., 2009). Therefore, it is widely recognized that in the peripheral circulation that increased vascular SR is important for improving endothelial function, irrespective of the stimulation method (Tinken et al., 2009, 2010). Interestingly, even in the cerebral circulation, a recent study demonstrated that increased SR induced by intermittent hypoxia enhanced hypercapnia‐induced dilatation in the ICA (Iwamoto et al., 2020). Thus, given that exercise increases CBF and cerebrovascular SR (Ogoh et al., 2021; Smith et al., 2019), it is reasonable to presume that exercise improves cerebral endothelial function.

However, in the present study, the interval HG did not alter cFMD (Figure 3) and cerebrovascular SR (Figure 2). Therefore, as a possible explanation for the lack of improvement in cFMD after HG, it was thought that this HG protocol may not be sufficient stimulation to increase cerebrovascular SR for improving cFMD. Currently, it is unclear why interval HG does not increase cerebrovascular SR; however, there is one possible mechanism related to *P*_ET_CO_2_ during interval HG. The previous study (Sakamoto et al., 2023) demonstrated that exercise‐induced elevation of *P*_ET_CO_2_ contributes to an increase in cerebrovascular SR. Actually, increased *P*_ET_CO_2_ and CBF enhance cerebrovascular SR (Ogoh et al., 2021; Smith et al., 2019), whereas unchanged *P*_ET_CO_2_ does not increase cerebrovascular SR during exercise (Sakamoto et al., 2021). The lack of change in *P*_ET_CO_2_ during HG in the present study may be attributed to the small muscle groups involved in the exercise. In the present study, ${\dot{V}}_{E}$ did not increase during interval HG (Table 1), indicating that metabolic activity is not large. Thus, to increase *P*_ET_CO_2_ and SR, and consequently improve the cerebral regulatory system, exercise with high metabolic demand may need to be used.

Another possible explanation for the lack of improvement in cFMD after HG may be attributed to an increase in sympathetic nervous activity after HG (Dawson et al., 2013). In peripheral circulation, it is suggested that FMD in the brachial artery is decreased by an exercise‐induced increase in sympathetic nerve activity (Atkinson et al., 2015). Similarly, in cerebral circulation, a decrease in hypercapnia‐induced dilatation in the ICA has been observed by the activation of sympathetic vasomotor activity caused by applying lower body negative pressure (Iwamoto et al., 2018a) and high‐intensity exercise (Sakamoto et al., 2021). Isometric exercise (HG) increases sympathetic nerve activity more than dynamic exercise (Lind, 1970). Thus, there is a possibility that improvement in cFMD during HG may be masked by an HG‐induced increase in sympathetic activity. In addition, it is known that cFMD can be altered by NO‐independent physiological factors, such as blood pressure (Hoiland et al., 2022). However, in the present study, arterial blood pressure was unchanged while measuring cFMD before and after interval HG (Tables 3 and 4). Thus, the effect of NO‐independent physiological factors on cFMD may be minimal. Importantly, the physiological properties of small muscle exercise such as HG that affects CBF regulation (small metabolism or high sympathetic nerve activity) should be considered when using small muscle exercise as exercise therapy for brain health.

Consistent with previous studies, the acute interval HG improved cognitive performance in young participants (Table 2), supporting that HG is effective in improving cognitive function. The results of the present study do not directly support that HG improves cognitive function in elderly and bedridden patients. However, it is well expected that HG is a useful exercise to improve the cognitive function of those individuals because young participants showed improved cognitive function with HG, despite their higher baseline cognitive function compared to populations at high risk of cognitive impairment (Salthouse, 2009). Importantly, acute exercise has been shown to improve cognitive function regardless of age (Hogan et al., 2013). On the other hand, it cannot be said that an adequate and effective HG protocol (i.e., intensity and duration) to improve cognitive performance has yet been established (Saito et al., 2021; Washio et al., 2021; Zhu et al., 2022). Indeed, we demonstrated that modifications of HG protocols that improve cognitive function, such as shortening the exercise duration in one set while maintaining the total workload, abolish the favorable effects on cognitive performance (Saito et al., 2021). It may be reasonable to presume that acute exercise that improves cognitive function can maintain chronic cognitive health through repeated performance. However, it is not fully understood how the acute effects of exercise on cognitive function determine chronic effects (Hashimoto et al., 2021), and thus caution is warranted in estimating the chronic effects from the present results.

On the other hand, some possible mechanisms of HG‐induced improvement in cognitive function are cerebral neural activity during cognitive tasks, psychological arousal, and an increase in blood lactate levels (Basso & Suzuki, 2017; Byun et al., 2014; Tsukamoto et al., 2016; Yanagisawa et al., 2010). However, the physiological mechanism remains unknown. Since knowing this physiological mechanism may be necessary to establish an adequate and effective HG protocol to improve brain health, further investigations are needed to identify it.

Some potential limitations of the present study should be considered. Since participants in the present study were all young healthy adults, these results cannot be directly applied to elderly, hypertensive and dementia patients. Indeed, interval HG training enhances peripheral vascular endothelial function in hypertensive patients with blunted peripheral vascular endothelial function (McGowan et al., 2006), but not in normotensive individuals (McGowan et al., 2007). Furthermore, the older adults have blunted shear‐mediated dilatation in the ICA compared with younger adults (Iwamoto et al., 2018b). Moreover, during exercise, the elderly exhibit different cerebrovascular and cardiovascular responses (e.g., enhance cerebrovascular resistance and exercise pressor reflex) than younger adults (Fisher et al., 2008; Smolander et al., 1998). Therefore, the cerebrovascular SR response during exercise and the effects of exercise on endothelial function in the cerebral vasculature may differ depending on participant characteristics similar to peripheral vascular endothelial function. Therefore, the effects of exercise on cerebral endothelial function should be investigated in the future in elderly adults and patients with hypertension, dementia, etc.

## 5 CONCLUSION

Acute interval HG did not alter cerebral endothelial function, although interval HG improved cognitive performance. These results suggest that improvement in cerebral endothelial function is not an essential mechanism responsible for acute HG‐induced cognitive improvement.

## AUTHOR CONTRIBUTIONS

Shotaro Saito and Shigehiko Ogoh conceptualized and designed the research; Shotaro Saito, Hironori Watanabe, and Shigehiko Ogoh performed experiments; Shotaro Saito and Hironori Watanabe analysed data; Erika Iwamoto and Shigehiko Ogoh interpreted the results of experiments; Shigehiko Ogoh prepared figures; Shotaro Saito and Shigehiko Ogoh drafted the manuscript; all authors edited and revised the manuscript. All authors have read and approved the final version of this manuscript and agree to be accountable for all aspects of the work in ensuring that questions related to the accuracy or integrity of any part of the work are appropriately investigated and resolved. All persons designated as authors qualify for authorship, and all those who qualify for authorship are listed.

## CONFLICT OF INTEREST

The author declare no conflicts of interest.

## Supporting information

Statistical Summary Document

## Data Availability

The datasets generated and analysed in the present study are available from the corresponding author upon reasonable request.
